# The Surgical and Therapeutic Activities of Non-Functional Pancreatic Neuroendocrine Tumors at a High-Volume Institution

**DOI:** 10.3390/cancers15071955

**Published:** 2023-03-24

**Authors:** Wu-Hu Zhang, Jun-Feng Xu, Yu-Heng Hu, Yi Qin, Jie Chen, Xian-Jun Yu, Xiao-Wu Xu, Shun-Rong Ji

**Affiliations:** 1 Department of Pancreatic Surgery, Fudan University Shanghai Cancer Center, Shanghai 200032, China; zhangwuhu@fudanpci.org (W.-H.Z.); xujunfeng@fudanpci.org (J.-F.X.); huyuheng@fudanpci.org (Y.-H.H.); qinyi@fudanpci.org (Y.Q.); yuxianjun@fudanpci.org (X.-J.Y.); xuxiaowu@fudanpci.org (X.-W.X.); 2 Center for Neuroendocrine Tumors, Fudan University Shanghai Cancer Center, Shanghai 200032, China; chen0jie@hotmail.com; 3 Shanghai Pancreatic Cancer Institute, Shanghai 200032, China; 4 Pancreatic Cancer Institute, Fudan University, Shanghai 200032, China; 5 Department of Oncology, Shanghai Medical College, Fudan University, Shanghai 200032, China; 6 Department of Head & Neck Tumors and Neuroendocrine Tumors, Fudan University Shanghai Cancer Center, Shanghai 200032, China

**Keywords:** pancreatic neuroendocrine tumors, surgery, treatment, survival analyses, prognosis

## Abstract

**Simple Summary:**

Non-functional pancreatic neuroendocrine tumors (NF-PanNETs) are a highly heterogeneous group of tumors with an increasing incidence. This study aimed to summarize the surgical and therapeutic activities of NF-PanNETs and to perform survival analyses at Fudan University Shanghai Cancer Center (FUSCC) over the past 15 years. We screened 1001 patients with neuroendocrine neoplasms treated at FUSCC, and 509 patients with NF-PanNETs from 2006 to 2020 were included. Time trend analyses revealed an increasing number of diagnosed and resected NF-PanNETs. Among three randomly divided periods, a significant decrease in the tumor size and a substantial increase in minimally invasive techniques were observed. In the subgroup of non-metastatic and resected NF-PanNETs, tumor size, positive lymph node, adjuvant treatment, and tumor grade were independent prognostic factors for recurrence-free survival. Microvascular invasion and tumor grade were independent prognostic factors for overall survival (OS). Notably, a malignant transformation from NET into neuroendocrine carcinoma was observed.

**Abstract:**

Background: This study aimed to summarize the surgical and therapeutic activities of non-functional pancreatic neuroendocrine tumors (NF-PanNETs) and perform survival analyses of a 15-year single-institutional cohort of NF-PanNETs. Methods: In total, 1001 patients with neuroendocrine neoplasms treated at Fudan University Shanghai Cancer Center were screened for inclusion, and 509 patients with NF-PanNETs from 2006 to 2020 were included. For time trend analyses, the 15-year study period was randomly divided into three periods. Survival analyses used the Kaplan–Meier method and Cox regression models. Results: The total number of resected NF-PanNETs increased over the 15-year study period, from 5 resections in 2006 to 94 resections in 2020. A significant decrease in the tumor size was observed, from a mean of 4.0 cm to 3.3 cm, and to 3.0 cm in the most recent period (*p* = 0.006). Minimally invasive techniques gradually increased from 3.5% to 12.9%, and finally to 46.4% in the most recent period (*p* < 0.001). In non-metastatic and resected tumors, the tumor size (*p* < 0.001), positive lymph node (*p* < 0.001), adjuvant treatment (*p* = 0.048), and tumor grade (*p* < 0.001) were independent prognostic factors for recurrence-free survival (RFS). The microvascular invasion (*p* = 0.024) and tumor grade (*p* = 0.013) were independent prognostic factors for overall survival (OS). A malignant transformation from NET into neuroendocrine carcinoma was observed. Conclusions: An increasing number of NF-PanNETs resection and minimally invasive surgery was shown. In non-metastatic and resected tumors NF-PanNETs, tumor size, positive lymph node, adjuvant treatment, and tumor grade were independent predictors of RFS. Microvascular invasion and tumor grade were independent prognostic factors for OS.

## 1. Introduction

Pancreatic neuroendocrine tumors (PanNETs) are a highly heterogeneous group of tumors characterized by various clinical manifestations, and their incidence is now estimated to be 0.48 in 100,000 in the United States [1] and 0.7 in 100,000 in Japan [2]. PanNETs can be further divided into functional and non-functional PanNETs (NF-PanNETs), which depends on whether the tumor secretes biologically active hormones and has hormone syndromes. Studies have shown that between 60% and 90% of PanNETs are non-functional [3,4]. Regarding the rarity and heterogeneity of NF-PanNETs, there are still some issues with the optimal management of some subgroups that have not reached a common understanding. Therapeutic strategies vary according to metastatic status, tumor size, and histology classification. Among them, surgery is still the only curative treatment for the management of NF-PanNETs.

At present, surgery is recommended for NF-PanNETs larger than 2 cm, having aggressive characteristics such as local invasion or lymph node metastases, and functional tumors [5]. However, whether small NF-PanNETs should be operated remains controversial. Although the three guidelines recommend a watchful waiting strategy for small tumors [6,7,8], the European Society for Medical Oncology (ESMO) guideline recommends a cautious attitude towards this method due to the short follow-up time and the lack of prospective studies [9]. Moreover, it has been reported that nearly 70–80% of small NF-PanNETs were surgically removed in clinical practice [10,11]. The aggressive approach for small NF-PanNETs in the real world may come from concerns about the malignant potential of small NF-PanNETs and the increased risk of metastases during follow-up [5,12]. In addition, surgery for NF-PanNETs patients with distant metastasis is increasingly recognized. It was reported that cytoreduction of more than 70% for patients with metastatic tumors can lead to a survival benefit [13,14,15].

Several classifications and staging and grading systems for PanNETs have emerged during the last decade. The World Health Organization (WHO) adopted and modified the European Neuroendocrine Tumor Society (ENETS) grading system in 2010 and 2017, respectively, in which PanNETs were defined as G1, G2, and G3 based on the cut-off point of the Ki67 proliferative index and mitotic rate [16,17,18]. Moreover, the ENETS and the American Joint Committee on Cancer (AJCC) tumor-node-metastasis (TNM) staging systems are also well-established [16,19,20]. Although grading and TNM staging systems have prognostic significance, there is no consensus on accurate prognostic factors for the prediction of survival and recurrence after PanNET resection, and hence they require further investigation.

Therefore, the present study summarized the surgical and therapeutic activities of NF-PanNETs at a high-volume tertiary referral center over the past 15 years. In particular, a survival analysis on each subgroup of NF-PanNETs was performed to summarize their respective survival. The following subgroups were discussed in detail: non-metastatic and resected tumors, metastatic tumors, small tumors ≤2 cm, second primary malignancy (SPM), and multifocal tumors. Notably, a dedifferentiation process from “PanNET G2” to poorly differentiated pancreatic neuroendocrine carcinoma (PanNEC) was observed in a patient, and the treatment for this patient was described.

## 2. Materials and Methods

### 2.1. Patient Population

**Figure 1:**
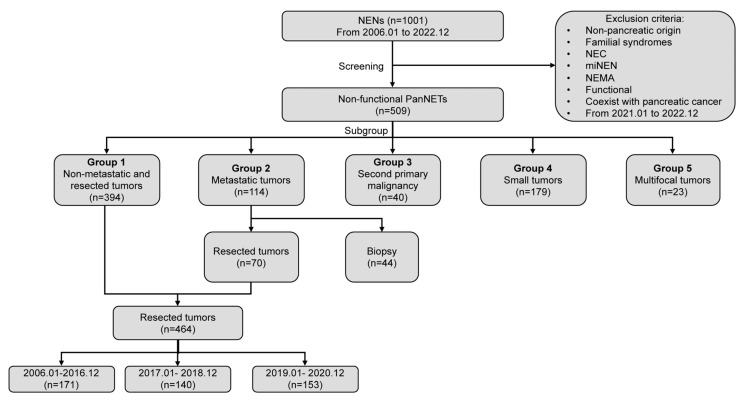
Flow diagram of patient selection. NENs: neuroendocrine neoplasms; NEC: neuroendocrine carcinoma; miNEN: mixed neuroendocrine-non-neuroendocrine neoplasms; NEMA: neuroendocrine microadenoma; PanNETs: pancreatic neuroendocrine tumors.

All patients who were treated for NENs attended histopathology at Fudan University Shanghai Cancer Center (FUSCC) between 1 January 2006 and 31 December 2022 where they were screened for inclusion. During this study period, a total of 1001 patients with NENs were screened. The exclusion criteria included non-pancreatic origin, familial syndromes, NEC, mixed neuroendocrine-non-neuroendocrine neoplasms (MiNEN), neuroendocrine microadenoma (NEMA), functional tumors, coexistence with pancreatic cancer, and new patients treated between January 2021 and to December 2022. Subsequently, 509 patients with NF-PanNETs were included and divided into different subgroups including non-metastatic and resected tumors (*n* = 394), metastatic tumors (*n* = 114, liver metastases; *n* = 111, peritoneal metastases; and other distant metastases, *n* = 3), tumors with SPM (*n* = 40), small tumors ≤2 cm (*n* = 179), and multifocal tumors (*n* = 23, Figure 1). Four hundred and sixty-four patients underwent resection, with a perioperative mortality of 0.2% (one case). For time trend analyses, the 15-year study period was randomly divided into three periods, with each containing a comparable study population (2006 to 2016, *n* = 171; 2017 to 2018, *n* = 140; and 2019 to 2020, *n* = 153. Supplementary Table S1).

Patient information, including demographic, clinical, operative, pathological, and radiological data were retrospectively retrieved from the medical record database. The pathological reports of all the patients were reviewed, and tumors were regraded based on the 2019 WHO classification and restaged according to the 8th edition AJCC TNM staging system of 2017. Patients were followed-up at 3- or 6-month intervals and underwent a physical examination, laboratory investigations, and at least one of the imaging techniques including contrast-enhanced computed tomography (CT), magnetic resonance imaging (MRI), and somatostatin receptor (SSR)-based positron-emission tomography/computed tomography (PET/CT). Patients were also contacted by phone if needed. The follow-up was updated in December 2022. Pancreatic surgery procedures included pancreaticoduodenectomy (PD), distal pancreatectomy (DP), total pancreatectomy (TP), enucleation (EN), or central pancreatectomy (CP). Margin status R0 and R1 were determined microscopically, and R2 was defined as incomplete resection. R2 resection, i.e., cytoreductive surgery, was performed for patients with metastatic tumors. Recurrence was diagnosed by radiologists or surgeons and was defined as local or distant, either in the pancreas or regional lymph nodes or in the liver, bone, intestine, or other distant metastases. Recurrence-free survival (RFS) was defined as the time from the date of curative resection to the date of recurrence or death. As for the group of metastatic tumors, progression-free survival (PFS) was defined as the time from the date of R2 resection or biopsy to the date of progression or death. Overall survival (OS) was defined as the time from the date of curative surgery or biopsy to the date of either death or the last follow-up. This retrospective study was approved by the institutional review board (IRB) of FUSCC, and informed consent was waived by the IRB due to the retrospective nature of this study.

### 2.2. Statistical Analyses

Statistical analyses were performed as previously described [21]. The final multivariable model used the forward stepwise selection method, and significant prognostic factors were presented with hazard ratios (HRs) and 95% confidence intervals (CIs). Time trend analyses were conducted using the Mantel–Haenszel and Kruskal–Wallis tests for categorical and continuous variables, respectively [22].

## 3. Results

### 3.1. Patient Characteristics and Surgical Activities

During the 15-year study period, a total of 509 NF-PanNETs were identified as the study cohort. Patient characteristics of the entire study cohort and each subgroup were listed in Table 1. The study cohort’s median age was 54 years, and 56.4% of patients were female. More than half of the tumors were located in the body or tail of the pancreas (53.8%), and the mean tumor size was 3.6 cm. The proportion of patients with positive lymph nodes, perineural invasion, and microvascular invasion were 20.9%, 19.2%, and 25.3%, respectively. The AJCC 8th TNM staging was distributed as follows: stage I—135 (26.5%), stage II—204 (40.1%), stage III—56 (11.0%), and stage IV—114 (22.4%). In total, 217 patients had G1 tumors (42.6%), 269 patients had G2 tumors (52.8%), and 23 patients had G3 tumors (4.5%). As for surgical activities, approximately 20% (95 patients) of NF-PanNETs underwent minimally invasive surgery (MIS). DP was the most frequent procedure (57.8%). In the 464 patients with resection, the rate of R0, R1, and R2 were 83.4%, 1.5%, and 15.1%, respectively.

### 3.2. Time Trends Analysis

**Figure 2:**
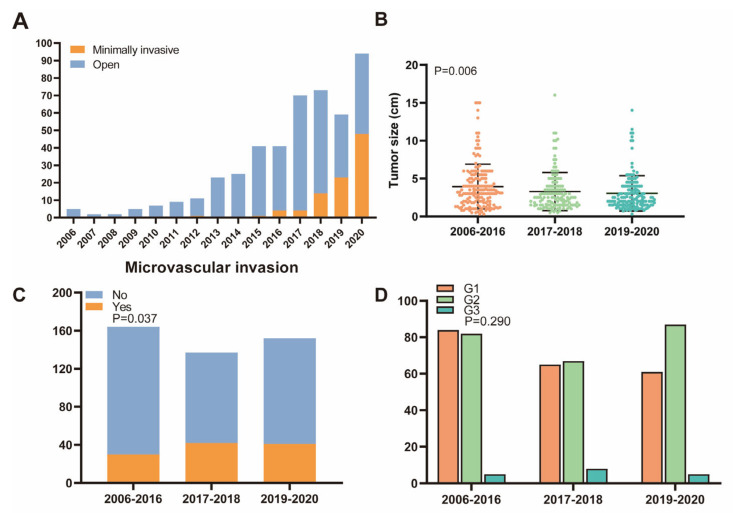
Time trends analysis of surgical activities and tumor characteristics over different periods. (**A**) The total number of NF-PanNETs resection and minimally invasive surgery increased over the 15-year study period. (**B**) Tumor size showed a significant decrease over different periods. (**C**) Microvascular invasion was significantly increased over different periods. (**D**) No significant difference was shown in WHO grades over different periods.

The total number of resected NF-PanNETs showed an increasing trend over the 15-year study period, from 5 resections in 2006 to 94 resections in 2020, with an annual growth rate of 5.93% (Figure 2A). There was no significant difference in the median age of the patients among the three subgroups of the 15-year study period (Supplementary Table S1). A substantial decrease in the tumor size was observed, from a mean of 4.0 cm until 2016, to 3.3 cm in the second period, and to 3.0 cm in the most recent period (*p* = 0.006, Figure 2B). Concerning surgical treatment, minimally invasive techniques gradually increased from 3.5% to 12.9%, and finally to 46.4% in the most recent period (*p* < 0.001). After 2020, the proportion of MIS procedures performed for NF-PanNET increased rapidly and reached about 90% in 2022. As for the pathology, the rate of microvascular invasion increased from 18.3% in the first period, to 30.7% in the second, and finally to 27.0% in the third (*p* = 0.037, Figure 2C). During the study period, the distribution of AJCC 8th staging (*p* = 0.485) and WHO grading (*p* = 0.290, Figure 2D) did not show a significant difference.

### 3.3. Non-Metastatic and Resected Tumors

Given that resection and metastatic tumors have a significant impact on survival, a specific subgroup of 394 patients with non-metastatic and resected tumors was performed for survival analyses (Table 1). After a mean follow-up of 46.7 months, the recurrence rate was 19.3% (76 patients). The estimated mean RFS was 116.2 months (95% CI: 104.4–128.0 months). In univariable analysis, the predictors for recurrence were tumor size (*p* < 0.001), positive lymph node (*p* < 0.001), perineural invasion (*p* = 0.001), microvascular invasion (*p* < 0.001), margin status (*p* = 0.001), adjuvant treatment (*p* < 0.001), and tumor grade (*p* < 0.001). In multivariable analysis, tumor size (HR = 4.975, 95% CI: 2.538–9.752, *p* < 0.001), positive lymph node (HR = 2.818, 95% CI: 1.628–4.879, *p* < 0.001), adjuvant treatment (HR = 1.840, 95% CI: 1.004–3.373, *p* = 0.048), and tumor grade (HR = 6.904, 95% CI: 2.994–15.916, *p* < 0.001) were independent prognostic factors for RFS (Table 2). The overall survival rate of this subgroup was 97.2% (383 patients). The estimated mean OS was over 15 years (186.2 months, 95% CI: 174.6–197.9 months). In univariable analysis, positive lymph node (*p* = 0.006), microvascular invasion (*p* = 0.012), adjuvant treatment (*p* = 0.015), and tumor grade (*p* < 0.001) were all statistically significant. In multivariable analysis, microvascular invasion (HR = 4.395, 95% CI: 1.215–15.905, *p* = 0.024) and tumor grade (HR = 7.718, 95% CI: 1.543–38.602, *p* = 0.013) were independent prognostic factors for OS (Table 2).

Regarding the therapeutic strategy of 394 patients with localized and resected NF-PanNETs, 2 patients with locally advanced tumors received neoadjuvant capecitabine and temozolomide (CAPTEM). In total, 39 patients (9.9%) received adjuvant treatment, of whom 32 patients received somatostatin analogs (SSAs), 3 patients received CAPTEM regimen, 2 patients received locoregional treatment, and the other 2 patients received traditional Chinese medicine treatment. Compared to patients without adjuvant treatment, the 39 patients who received adjuvant treatment had a larger mean tumor size (4.2 cm vs. 3.0 cm), higher R1 rate (5.1% vs. 1.4%), higher lymph node positivity (51.3% vs. 9.9%), higher rate of perineural invasion (37.8% vs. 10%), higher rate of microvascular invasion (59.5% vs. 14.6%), and a higher rate of G2 or G3 tumors (79.5% vs. 45.6%).

### 3.4. Metastatic Tumors

**Figure 3:**
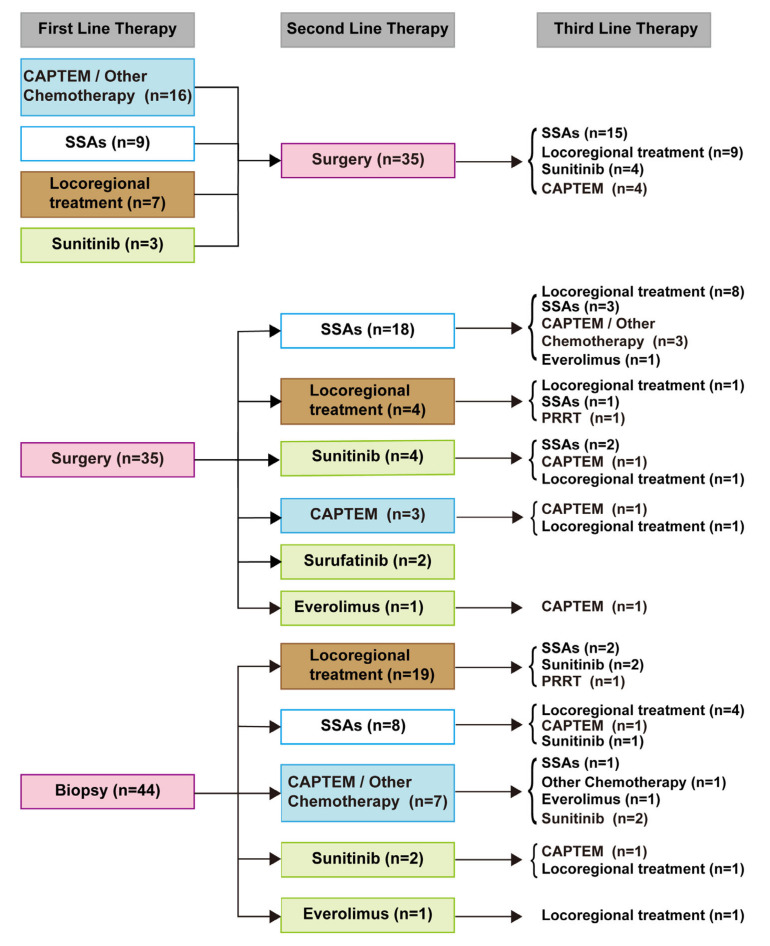
FUSCC treatment flowchart for patients with metastatic NF-PanNETs. A few patients received more than three lines of therapy, and some up to seven. CAPTEM: capecitabine and temozolomide; SSAs: somatostatin analogs; PRRT: peptide receptor radionuclide therapy. Locoregional treatment includes microwave ablation of the liver, transarterial chemoembolization, and transarterial embolization. Other chemotherapy includes capecitabine, carboplatin, fluorouracil, irinotecan, and oxaliplatin.

Patient characteristics of the 114 metastatic tumors were listed in Table 1. The rate of progression was 76.3% (87 patients). The estimated mean PFS was 20.2 months (95% CI: 16.2–24.2 months). The overall survival rate of this subgroup was 88.6% (101 patients). The estimated mean OS was 115.9 months (95% CI: 102.0–129.7 months). Treatment patterns were summarized in Figure 3. Treatments were categorized as (1) SSAs; (2) CAPTEM or other chemotherapy (e.g., capecitabine, carboplatin, fluorouracil, irinotecan, and oxaliplatin); (3) locoregional treatment (microwave ablation of the liver, transarterial chemoembolization, and transarterial embolization); (4) surgery or biopsy; (5) targeted therapy (e.g., sunitinib, everolimus, and surufatinib); and (6) peptide receptor radionuclide therapy (PRRT). In total, 70 patients underwent resection and 44 patients underwent biopsy. Notably, 35 patients received treatment before cytoreductive surgery, and CAPTEM-based chemotherapy was the most frequently used. Among the 35 patients who received post-operative treatment, SSAs were the most common form of treatment. For patients who underwent biopsy, locoregional treatment was the most common treatment.

### 3.5. Tumors with Second Primary Malignancy

Out of the 509 NF-PanNETs, 40 patients (7.9%) presented with SPM. The characteristics of the subgroup were described (Table 1). The most frequent SPM were gynecological tumors (17.5%) followed by colorectal tumors (15.0%) and gastrointestinal stromal tumors (15.0%) (Table 3). The recurrence rate was 30.0% (12 patients). The estimated mean RFS of this subgroup was 65.2 months (95% CI: 52.3–78.2 months). The overall survival rate was 92.5% (37 patients). The estimated mean OS was 86.2 months (95% CI: 79.0–93.5 months). The SPM was synchronous in 17 cases (42.5%), metachronous in 10 cases (25.0%), and previously diagnosed in 13 cases (32.5%). Among the 10 metachronous cases, 2 patients (20.0%) previously received SSAs, and 1 (10.0%) had CAPTEM, while others did not receive treatment for primary NF-PanNETs before the SPM occurrence. Among the 13 patients with antecedent SPM before NF-PanNETs occurrence, 5 patients (38.5%) previously received chemotherapy, and 2 (15.4%) had chemotherapy and targeted therapy.

### 3.6. Small Tumors ≤2 cm

Of the 509 NF-PanNETs, 179 patients had small tumors ≤2 cm (Figure 1). The median age was 55 years, and the male-to-female ratio was 1:1.56 (Table 1). In total, 47 patients (26.3%) had tumors located in the head of the pancreas, and 9 patients (5.0%) had multifocal tumors. The mean tumor size was 1.4 cm. A total of 154 patients (86.0%) had tumors ≥1 cm, and 25 patients (14.0%) had tumors of less than 1 cm. Among the 179 patients, 176 underwent surgery, and 3 patients received a biopsy. Overall, 52 patients (29.5%) received PD, 92 (52.3%) received DP, 26 (14.8%) received EN, and 6 (3.4%) received CP. Furthermore, 122 patients (68.2%) had G1, 54 (30.2%) had G2, and 3 (1.7%) had G3. As for metastatic status, 11 patients (6.1%) had liver metastases and 12 patients (6.7%) had positive lymph nodes. The estimated mean RFS was 110.5 months (95% CI: 100.3–120.6 months), and the estimated mean OS was 195.4 months (95% CI: 190.5–200.4 months. Supplementary Figure S1. The recurrence rate was 9.5% (17 patients), and the overall survival rate was 98.9% (177 patients). Patients with small tumors had significantly longer RFS and OS than tumors >2 cm (*p* < 0.001 and *p* = 0.020, respectively), Supplementary Figure S1.

### 3.7. Multifocal Tumors

Excluding patients with familial syndromes, multifocal tumors were observed in 23 patients (4.5%) of the 509 NF-PanNETs (Table 1). The median age was 52 years, and the mean tumor size was 4.1 cm. Among the 23 patients, 22 received surgery, and one patient received a biopsy. Overall, 8 patients (36.4%) received PD, 10 (45.5%) received DP, and 4 (18.2%) received TP. In total, 10 patients (43.5%) had G1, 12 (52.2%) had G2, and 1 (4.3%) had G3. Notably, in a patient with two primary tumors, one tumor was G1 with a Ki67 of 2% and the other was G2 with a Ki67 of 3%. Other multifocal patients had the same tumor grade. For patients with multifocal NF-PanNETs before 2015, the pathological reports only included the Ki67 index and the grade of one tumor. The estimated mean RFS of this subgroup was 81.4 months (95% CI: 63.6–99.2 months), and the estimated mean OS was 177.1 months (95% CI: 165.6–188.6 months). The recurrence rate was 21.7% (5 patients), and the overall survival rate was 95.7% (22 patients). There was no significant difference in RFS and OS between patients with multifocal tumors and those with only one tumor.

### 3.8. Transformation from NET to NEC

**Figure 4:**
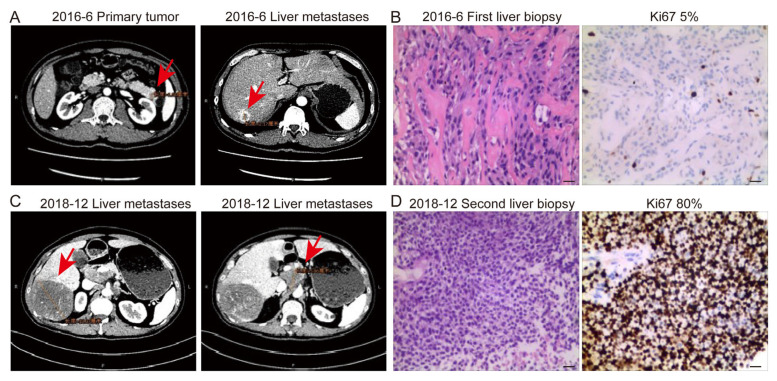
Contrast-enhanced computed tomography showed primary tumor and liver metastases (red arrows) at initial diagnosis and progression. (**A**,**C**). Hematoxylin and eosin staining and immunohistochemistry staining of Ki67 of each lesion. Scale bar indicates 20 μm. The Ki67 indexes of the first and second liver biopsies were 5% (**B**) and 80% (**D**), respectively.

In the present series, a 41-year-old man transformed in terms of his classification, from “NET-G2” to poorly differentiated “NEC” over a time period of 2 years (Figure 4). At initial diagnosis, CT showed a 1.8 cm tumor in the tail of the pancreas and multiple liver metastases. The patient underwent the first liver biopsy in June 2016 and was diagnosed as having “NET-G2” with a Ki67 index of 5%. After treatment with SSAs, progression was observed in imaging, and a second liver biopsy was performed in December 2018. The poorly-differentiated NEC was confirmed by pathology with a Ki67 index of 80%, and a combination of etoposide and cisplatin was administered. However, the tumors progressed rapidly and were resistant to treatment. The patient died in October 2019.

## 4. Discussion

To the best of our knowledge, this retrospective study represents the largest single-institution cohort of NF-PanNETs operated and treated in China. By encompassing more than 15 years, this study screened 1001 patients with NENs and ultimately identified 509 NF-PanNETs patients as the study cohort. This study depicted the evolution of surgery and treatment of NF-PanNETs, and survival analyses were performed for each subgroup.

The time trend analyses showed an increasing number of resections of NF-PanNETs, from 5 resections in 2006 to 94 resections in 2020, with an annual growth rate of 5.93%. Moreover, the number of MIS gradually increased, from 0 in 2006 to 48 cases in 2020, with an annual growth rate of 3.2%. This trend was consistent with other reported surgical cohorts [22,23]. Our institute established an MIS center in 2018, and since then, the proportion of MIS has gradually increased. Significantly, the proportion of MIS performed for NF-PanNETs reached nearly 90% in 2022, indicating that our institution has become one of the largest and most specialized MIS centers for PanNETs in China. The main reason for the increase in MIS was the improvement of doctors’ technical skills and a better understanding of the biological characteristics of NF-PanNETs. Due to the improvement of people’s health consciousness, the increased utilization of high-resolution imaging, and the fact that NF-PanNETs were originally considered an indication for surgery, it was more common for patients to incidentally discovere and resect small NF PanNETs. Therefore, the trend of tumor size showed a significant decrease in the three different study periods, namely from a mean of 4.0 cm to 3.0 cm.

In 394 patients with non-metastatic and resected tumors, survival analysis showed that tumor size, positive lymph node, adjuvant treatment, and tumor grade were independent prognostic factors for RFS. Microvascular invasion and tumor grade were independent prognostic factors for OS. NF-PanNETs were a highly heterogenous group of tumors; though relatively indolent, some studies revealed that the overall recurrence rate after resection could be as high as 13.7% to 25.5% [24,25,26,27]. It has been reported that tumor size, tumor grade, margin status, positive lymph node, perineural and microvascular invasion, and pancreatic duct dilatation were risk factors for the recurrence of PanNENs [26,28,29,30]. Therefore, it was necessary to fully evaluate whether the resected patients had the above risk factors and select the appropriate adjuvant treatment. As for therapeutic activities, 39 out of the 394 patients received adjuvant treatment. They had a larger tumor size, higher R1 rate, higher lymph node positivity, higher rate of perineural and microvascular invasion, and higher rate of G2 or G3 tumors than those who did not receive adjuvant treatment.

We also summarized treatment for metastatic tumors. Among the 114 metastatic patients with NF-PanNETs, 70 patients received cytoreductive surgery, and the other 44 patients received biopsy and systemic treatment. For treatment patterns, CAPTEM was the most frequently used among 35 patients who received treatment before cytoreductive surgery. SSAs were the most common treatment among the 35 patients who received postoperative treatment. The most common treatment for patients who underwent biopsy was locoregional treatment. The introduction of a multidisciplinary approach made the treatment of metastatic patients more standardized and personalized.

Our analysis showed that nearly 8% of NF-PanNETs patients presented with SPM. It has been reported that the rate of NENs cases with SPM was 9.8–55% [31,32,33], compared to only 1–3% of cases with other tumors [31]. This indicated a tumor susceptibility of SPM for PanNETs, even though there was no direct impact on overall survival. Therefore, when there was a tumor at another site, the possibility of SPM should be considered in addition to metastatic diseases. Imaging techniques such as CT, MRI, SSR-based PET/CT, or FDG-PET/CT were required to assist in the diagnosis of cases of PanNET suspected of SPM. Based on previous literature, SPM was mainly localized in the gastrointestinal and genitourinary tracts [33]. In the present series, the most frequent SPM was gynecological tumors, followed by colorectal tumors and gastrointestinal stromal tumors. Of note, in this NF-PanNET cohort, the SPM was antecedent in 32.5%, synchronous in 19.2%, and metachronous in 43.2% of cases. The mechanisms of this possible association were still far from being understood.

Surgery was usually recommended for NF-PanNETs >2 cm or functional tumors [6,7,8]. Debulking surgery was also recommended for metastatic tumors. However, it was still controversial whether to perform surgery or observation for the treatment of small NF-PanNETs. Some reports emphasized that small tumors could also behave aggressively and that clinical outcomes would be improved after resection [10,34]. While some other studies suggested that many small NF-PanNETs were biologically indolent, some did not progress over time and thus could be safely observed [35]. ASPEN and PANDORA were two prospective and multicenter studies designed to explore the optimal management for patients with NF-PanNETs ≤2 cm [36,37,38]. The results showed that the non-surgical strategy seemed to be safe and feasible. While the reasons for surgery were the patient’s preference, younger age, tumor size >1 cm, and main pancreatic duct dilation. In our study, 179 patients had tumors smaller than 2 cm. Among them, 176 cases received surgery, and the other 3 cases received biopsy and systemic treatment due to liver metastases. A limitation of this study was the lack of patients with active surveillance for a small NF-PanNET. Thus, it was impossible to compare the clinical outcomes of the surveillance and surgery. Among 179 patients with small NF-PanNETs, 25 patients with tumors <1 cm underwent surgery. Their strong surgical preference and the fear of tumor recurrence were the main reasons for choosing surgery.

Multifocal tumors were found in 23 (4.5%) patients, but no significant difference in RFS and OS was shown between patients with multifocal and unifocal tumors. Notably, a patient with two heterogenous tumors was observed, while a tumor was G1 with a Ki67 of 2%, and the other was G2 with a Ki67 of 3%. The highly heterogeneous characteristic of NF-PanNETs required us to emphasize the importance of pathological examinations of each tumor in multifocal PanNETs. In clinical practice, detailed examinations were required before surgery to avoid missing multifocal tumors. Additionally, patients with multifocal tumors should be excluded from hereditary diseases by imaging, including chest/abdominal/pelvic CT or MRI scans, endoscopic ultrasound, SSR-based PET/CT, or FDG-PET/CT.

Notably, a patient transformed from “PanNET G2” into poorly differentiated “NEC” was observed over a period of 2 years, indicating this transformation was indeed present in real-world clinical practice. Similarly, a 10-gene panel clustering analysis, derived from second-generation sequencing data of 48 GEP-NENs, preliminarily supported the hypothesis that a subgroup of GEP-NEC cells might evolve from the pre-existing well-differentiated NETs [39]. This phenomenon was interesting and could be an excellent clinical model for investigating the biological characteristics of PanNENs. Concerning animal models, Yamauchi et al. [40] reported that the simultaneous induction of the p53 mutation and Rb gene deletion could lead to the high-grade transformation of pancreatic islet cells, from well-differentiated PanNET with a Ki67 index of 2.7% to aggressive PanNET with a Ki67 index of 24.7%. In addition, high-grade transformation in liver metastases compared with primary tumors was found in our previous study, and the *TP53* mutation might contribute to the high-grade transformation [21]. The mechanism of high-grade transformation or malignant transformation from NET to NEC is still far from clear and requires further exploration.

Several limitations existed in the current study. Firstly, all retrospective studies have inherent limitations. Secondly, the median follow-up time of this study was relatively short, which might underestimate the overall survival time. Thirdly, this study lacked follow-up for patients with an active surveillance of small NF-PanNET. Since they represented a crucial subgroup to depict the natural history of the tumor, we could not compare the clinical outcomes of the surveillance and surgery. Finally, complications in surgical patients were not described in this study.

## 5. Conclusions

This study summarized the surgical and therapeutic activities of 509 NF-PanNETs in a high-volume tertiary referral center over the past 15 years. An increasing number of diagnosed and resected NF-PanNETs were shown. The improvement of doctors’ technical skills and a better understanding of the biological characteristics of NF-PanNETs led to an increase in MIS. The improvement in people’s health awareness and increased utilization of high-resolution imaging resulted in a significant decrease in the mean size of the resected tumors. At long-term follow-up, the clinical outcomes of non-metastatic and resected NF-PanNETs were extremely favorable. Tumor size, positive lymph node, adjuvant treatment, and tumor grade were reliable predictors for RFS. Microvascular invasion and tumor grade were independent prognostic factors for OS.

## Figures and Tables

**Table 1:** Demographics and clinical characteristics of the NF-PanNETs cohort and each subgroup.

Characteristics	Study Cohort (*n* = 509)	Group 1 (*n* = 394)	Group 2 (*n* = 114)	Group 3 (*n* = 40)	Group 4 (*n* = 179)	Group 5 (*n* = 23)
	No. (%)					
**Gender**						
Male	222 (43.6)	169 (42.9)	53 (46.5)	13 (32.5)	70 (39.1)	12 (52.2)
Female	287 (56.4)	225 (57.1)	61 (53.5)	27 (67.5)	109 (60.9)	11 (47.8)
**Median Age, years**	54	55	50.0	56.5	55.0	52.0
**Tumor size, cm**						
Mean (SD)	3.6 (2.7)	3.1 (2.4)	5.4 (3.0)	3.8 (2.2)	1.4 (0.4)	4.1 (3.2)
**Location**						
Head	147 (28.9)	118 (29.9)	28 (24.6)	8 (20.0)	47 (26.3)	0 (0.0)
Neck	65 (12.8)	58 (14.7)	7 (6.1)	4 (10.0)	34 (19.0)	0 (0.0)
Body	74 (14.5)	62 (15.7)	12 (10.5)	5 (12.5)	33 (18.4)	0 (0.0)
Tail	82 (16.1)	48 (12.2)	34 (29.8)	5 (12.5)	22 (12.3)	0 (0.0)
Body-Tail	118 (23.2)	87 (22.1)	31 (27.2)	13 (32.2)	34 (19.0)	0 (0.0)
Multifocal	23 (4.5)	21 (5.3)	2 (1.8)	5 (12.5)	9 (5.0)	23 (100.0)
**Lymph node positive**	*n* = 470		*n* = 76		*n* = 176	*n* = 22
Yes	98 (20.9)	55 (14.0)	43 (56.6)	9 (22.5)	12 (6.8)	6 (27.3)
No	372 (79.1)	339 (86.0)	33 (43.4)	31 (77.5)	164 (93.2)	16 (72.7)
**Perineural invasion**	*n* = 470	*n* = 387	*n* = 71	*n* = 39	*n* = 176	*n* = 22
Yes	88 (19.2)	49 (12.7)	39 (54.9)	4 (10.3)	17 (9.7)	8 (36.4)
No	370 (80.8)	338 (87.3)	32 (45.1)	35 (89.7)	159 (90.3)	14 (63.6)
**Microvascular invasion**	*n* = 458	*n* = 387	*n* = 71	*n* = 39	*n* = 176	*n* = 22
Yes	116 (25.3)	73 (18.9)	43 (60.6)	13 (33.3)	20 (11.4)	4 (18.2)
No	342 (74.7)	314 (81.1)	28 (39.4)	26 (66.7)	156 (88.6)	18 (81.8)
**Metastases**	*n* = 114			*n* = 5	*n* = 11	*n* = 2
Liver	111 (97.4)	0 (0.0)	111 (97.4)	5 (100.0)	11 (100.0)	2 (100.0)
Peritoneum/others	3 (2.6)	0 (0.0)	3 (2.6)	0 (0.0)	0 (0.0)	0 (0.0)
**Surgical approach**	*n* = 464		*n* = 70		*n* = 176	*n* = 22
Open	369 (79.5)	305 (77.4)	64 (91.4)	34 (85.0)	128 (72.7)	19 (86.4)
Minimally invasive	95 (20.5)	89 (22.6)	6 (8.6)	6 (15.0)	48 (27.3)	3 (13.6)
**Surgical procedure**	*n* = 464	*n* = 503	*n* = 70		*n* = 176	*n* = 22
PD	141 (30.4)	129 (32.7)	12 (17.1)	12 (30.0)	52 (29.5)	8 (36.4)
DP	268 (57.8)	213 (54.1)	55 (78.6)	25 (62.5)	92 (52.3)	10 (45.5)
TP	10 (2.2)	8 (2.0)	2 (2.9)	1 (2.5)	0 (0.0)	4 (18.2)
EN	36 (7.8)	35 (8.9)	1 (1.4)	1 (2.5)	26 (14.8)	0 (0.0)
CP	9 (1.9)	9 (2.3)	0 (0.0)	1 (2.5)	6 (3.4)	0 (0.0)
**Margin status**	*n* = 464		*n* = 70		*n* = 176	*n* = 22
R0	387 (83.4)	387 (98.2)	0 (0.0)	35 (87.5)	167 (94.9)	21 (95.5)
R1	7 (1.5)	7 (1.8)	0 (0.0)	0 (0.0)	1 (0.6)	0 (0.0)
R2	70 (15.1)	0 (0.0)	70 (100.0)	5 (12.5)	8 (4.5)	1 (4.5)
**AJCC 8th TNM stage**						
I	135 (26.5)	135 (34.3)	0 (0.0)	6 (15.0)	134 (74.9)	4 (17.4)
II	204 (40.1)	203 (51.5)	0 (0.0)	23 (57.5)	25 (14.0)	12 (52.2)
III	56 (11.0)	56 (14.2)	0 (0.0)	6 (15.0)	9 (5.0)	5 (21.7)
IV	114 (22.4)	0 (0.0)	114 (100.0)	5 (12.5)	11 (6.1)	2 (8.7)
**WHO classification**						
G1	217 (42.6)	201 (51.0)	15 (13.2)	13 (32.5)	122 (68.2)	10 (43.5)
G2	269 (52.8)	184 (46.7)	85 (74.6)	26 (65.0)	54 (30.2)	12 (52.2)
G3	23 (4.5)	9 (2.3)	14 (12.3)	1 (2.5)	3 (1.7)	1 (4.3)

SD: standard deviation; PD: pancreaticoduodenectomy; DP: distal pancreatectomy; TP: total pancreatectomy; EN: enucleation; CP: central pancreatectomy; AJCC: American Joint Committee on Cancer; WHO: World Health Organization.

**Table 2:** Univariable and multivariable analysis of factors for recurrence-free survival and overall survival in patients with non-metastatic and resected NF-PanNETs.

Factors	Recurrence-Free Survival				Overall Survival			
	Univariable		Multivariable		Univariable		Multivariable	
	HR (95% CI)	*p*-Value	HR (95% CI)	*p*-Value	HR (95% CI)	*p*-Value	HR (95% CI)	*p*-Value
Gender: male vs. female	0.863 (0.547–1.360)	0.525			1.249 (0.366–4.268)	0.723		
Age: <55 vs. ≥55 years	1.283 (0.813–2.025)	0.285			1.401 (0.408–4.814)	0.593		
Tumor size: <2.5 vs. ≥2.5 cm	6.120 (3.142–11.919)	**<0.001**	4.975 (2.538–9.752)	**<0.001**	7.662 (0.974–60.291)	0.053		
Tumor location: head vs. neck/body/tail	1.069 (0. 657–1.738)	0.789			1.281 (0.340–4.831)	0.714		
Lymph node positive: no vs. yes	4.448 (2.745–7.206)	**<0.001**	2.818 (1.628–4.879)	**<0.001**	5.284 (1.605–17.398)	**0.006**		
Perineural invasion: no vs. yes	2.608 (1.527–4.455)	**0.001**			3.446 (0.861–13.782)	0.080		
Microvascular invasion: no vs. yes	3.317 (2.058–5.345)	**<0.001**			4.958 (1.425–17.250)	**0.012**	4.395 (1.215–15.905)	**0.024**
Margin status: R0 vs. R1	4.963 (1.997–12.331)	**0.001**			4.794 (0.606–37.939)	0.138		
Adjuvant treatment: no vs. yes	3.643 (2.178–6.093)	**<0.001**	1.840 (1.004–3.373)	**0.048**	4.587 (1.338–15.730)	**0.015**		
WHO classification: G1/G2 vs. G3	12.357 (5.810–26.280)	**<0.001**	6.904 (2.994–15.916)	**<0.001**	16.665 (4.225–65.732)	**<0.001**	7.718 (1.543–38.602)	**0.013**

HR: hazard ratio; CI: confidence interval; WHO: World Health Organization.

**Table 3:** Characteristics of the second primary malignancy cohort.

Characteristics	SPM Cohort (*n* = 40)
	No. (%)
**Site**	
Gynecological tumors	7 (17.5)
Colorectal	6 (15.0)
Gastrointestinal stromal tumor	6 (15.0)
Breast	4 (10.0)
Thyroid	4 (10.0)
Endocrine system	4 (10.0)
Lung	2 (5.0)
Liver	2 (5.0)
Esophagus	1 (2.5)
Stomach	1 (2.5)
Prostate	1 (2.5)
Testis	1 (2.5)
Breast + lung	1 (2.5)
**Timing**	
Synchronous	17 (42.5)
Metachronous Antecedent	10 (25.0) 13 (32.5)
**Previous therapy for PanNETs in metachronous SPM**	*n* = 10
SSAs	2 (20.0)
CAPTEM	1 (10.0)
No	7 (70.0)
**Therapy for antecedent SPM administered before PanNETs occurrence**	*n* = 13
Chemotherapy	5 (38.5)
Chemotherapy + Targeted therapy	2 (15.4)
No	6 (46.1)

SPM: second primary malignancy; PanNETs: pancreatic neuroendocrine tumors; SSAs: somatostatin analogs; CAPTEM: capecitabine and temozolomide.

## Data Availability

Restrictions apply to the availability of some or all the data generated or analyzed during this study to preserve patient confidentiality or because they were used under license. The corresponding author will detail the restrictions and any conditions under which access to some data may be provided upon request.

## Supplementary Materials

The following supporting information can be downloaded at: https://www.mdpi.com/article/10.3390/cancers15071955/s1, Figure S1: Kaplan-Meier survival curves of patients with small tumors. (A) Recurrence-free survival curve estimated by Kaplan-Meier methods. (B) Overall survival curve estimated by Kaplan-Meier methods. (C,D) Kaplan-Meier curves depicting recurrence-free survival and overall survival for patients with tumors >2 cm or ≤2 cm; Table S1: Demographics and clinical characteristics of the non-metastatic and resected NF-PanNETs cohort during three periods.
