# Supplementary material for: The Surgical and Therapeutic Activities of Non-Functional Pancreatic Neuroendocrine Tumors at a High-Volume Institution
Source: Cancers (Basel). 2023 Mar 24;15(7):1955. doi: 10.3390/cancers15071955 (PMC10093673; doi:10.3390/cancers15071955)
Supplement: Supplementary file 1 [file cancers-15-01955-s001.zip › Supplementary material.pdf]

Figure S1. Kaplan-Meier survival curves of patients with small tumors. (A) Recurrence-free survival curve estimated by Kaplan-Meier methods. (B) Overall survival curve estimated by Kaplan-Meier methods. (C-D) Kaplan-Meier curves depicting recurrence-free survival and overall survival for patients with tumors >2 cm or ≤2 cm.

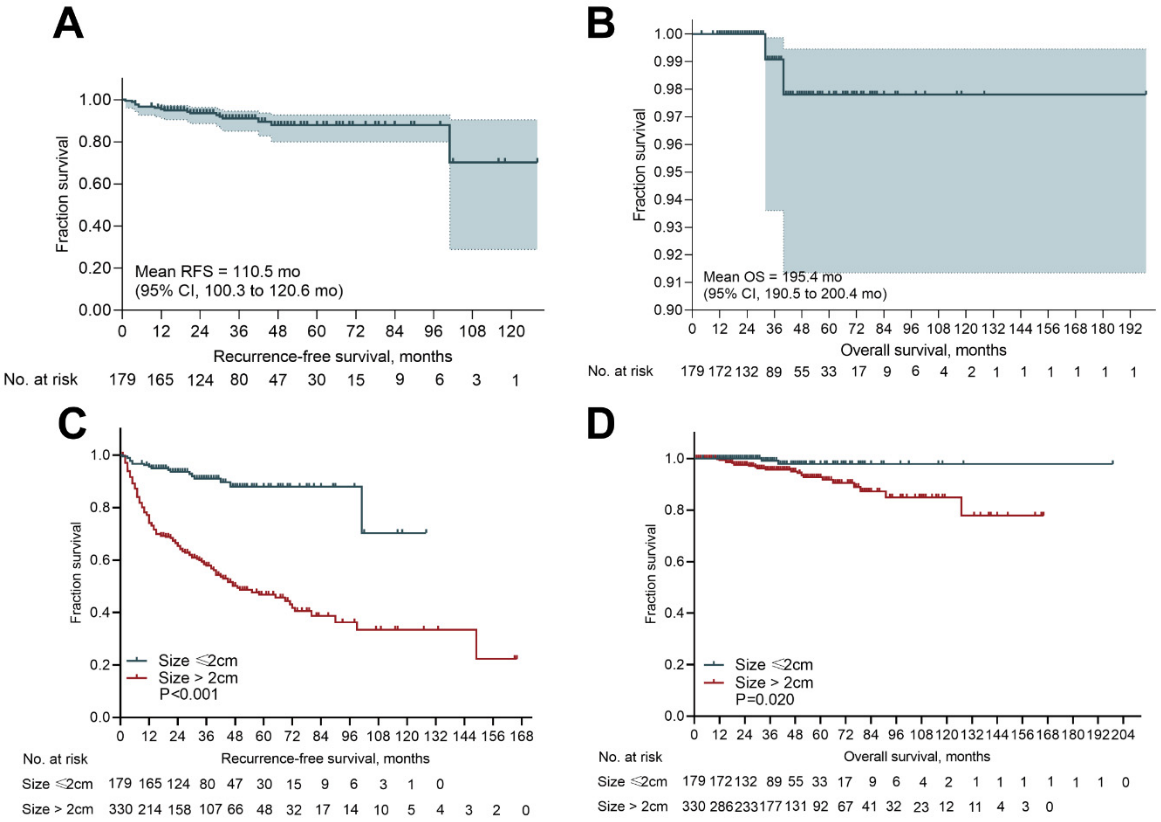

Table S1. Demographics and clinical characteristics of the non-metastatic and resected NF-PanNETs cohort during three periods

Characteristics	2006-2016 (n=171)	2017-2018 (n=140)	2019-2020 (n=153)	*P*-value
	No. (%)	No. (%)	No. (%)	
**Gender**				0.863
Male	72 (42.1)	61 (43.6)	69 (45.1)	
Female	99 (57.9)	79 (56.4)	84 (54.9)	
**Age, years, median**	54	54	53	0.732
**Tumor size, cm, Mean (SD)**	4.0 (3.0)	3.3 (2.5)	3.0 (2.3)	**0.006**
**Location**				0.332
Head	41 (24.0)	49 (35.0)	40 (26.1)	
Neck	25 (14.6)	17 (12.1)	20 (13.1)	
Body	24 (14.0)	22 (15.7)	22 (14.4)	
Tail	34 (19.9)	15 (10.7)	19 (12.4)	
Body-Tail	39 (22.8)	32 (22.9)	43 (28.1)	
Multifocal	8 (4.7)	5 (3.6)	9 (5.9)	
**Lymph node positive**				0.374
Yes	35 (20.5)	33 (23.6)	26 (17.0)	
No	136 (79.5)	107 (76.4)	127 (83.0)	
**Perineural invasion**	n=164	n=137	n=152	0.123
Yes	28 (17.1)	33 (24.1)	23 (15.1)	
No	136 (82.9)	104 (75.9)	129 (84.9)	
**Microvascular invasion**	n=164	n=137	n=152	**0.037**
Yes	30 (18.3)	42 (30.7)	41 (27.0)	
No	134 (81.7)	95 (69.3)	111 (73.0)	
**Metastases**				0.506
Yes	29 (17.0)	22 (15.7)	19 (12.4)	
No	142 (83.0)	118 (84.3)	134 (87.6)	
**Surgical approach**				**<0.001**
Open	165 (96.5)	122 (87.1)	82 (53.6)	
Minimally invasive	6 (3.5)	18 (12.9)	71 (46.4)	
**Surgical procedure**				0.258
PD	43 (25.1)	52 (37.1)	46 (30.1)	
DP	102 (59.6)	72 (51.4)	94 (61.4)	
TP	6 (3.5)	2 (1.4)	2 (1.3)	
EN	15 (8.8)	11 (7.9)	10 (6.5)	
CP	5 (2.9)	3 (2.1)	1 (0.7)	

**Margin status**				0.090
R0	140 (81.9)	113 (80.7)	134 (87.6)	
R1	2 (1.2)	5 (3.6)	0 (0.0)	
R2	29 (17.0)	22 (15.7)	19 (12.4)	
**AJCC 8th TNM stage**				0.485
I	43 (25.1)	40 (28.6)	52 (34.0)	
II	80 (46.8)	57 (40.7)	66 (43.1)	
III	19 (11.1)	21 (15.0)	16 (10.5)	
IV	29 (17.0)	22 (15.7)	19 (12.4)	
**WHO classification**				0.290
G1	84 (49.1)	65 (46.4)	61 (39.9)	
G2	82 (48.0)	67 (47.9)	87 (56.9)	
G3	5 (2.9)	8 (5.7)	5 (3.3)	

Note: SD: standard deviation; PD: pancreaticoduodenectomy; DP: distal pancreatectomy; TP: total pancreatectomy; EN: enucleation; CP: central pancreatectomy; AJCC: American Joint Committee on Cancer; WHO: World Health Organization.
