# Supplementary figures and images for: The Surgical and Therapeutic Activities of Non-Functional Pancreatic Neuroendocrine Tumors at a High-Volume Institution
Source: Cancers (Basel). 2023 Mar 24;15(7):1955. doi: 10.3390/cancers15071955 (PMC10093673; doi:10.3390/cancers15071955)

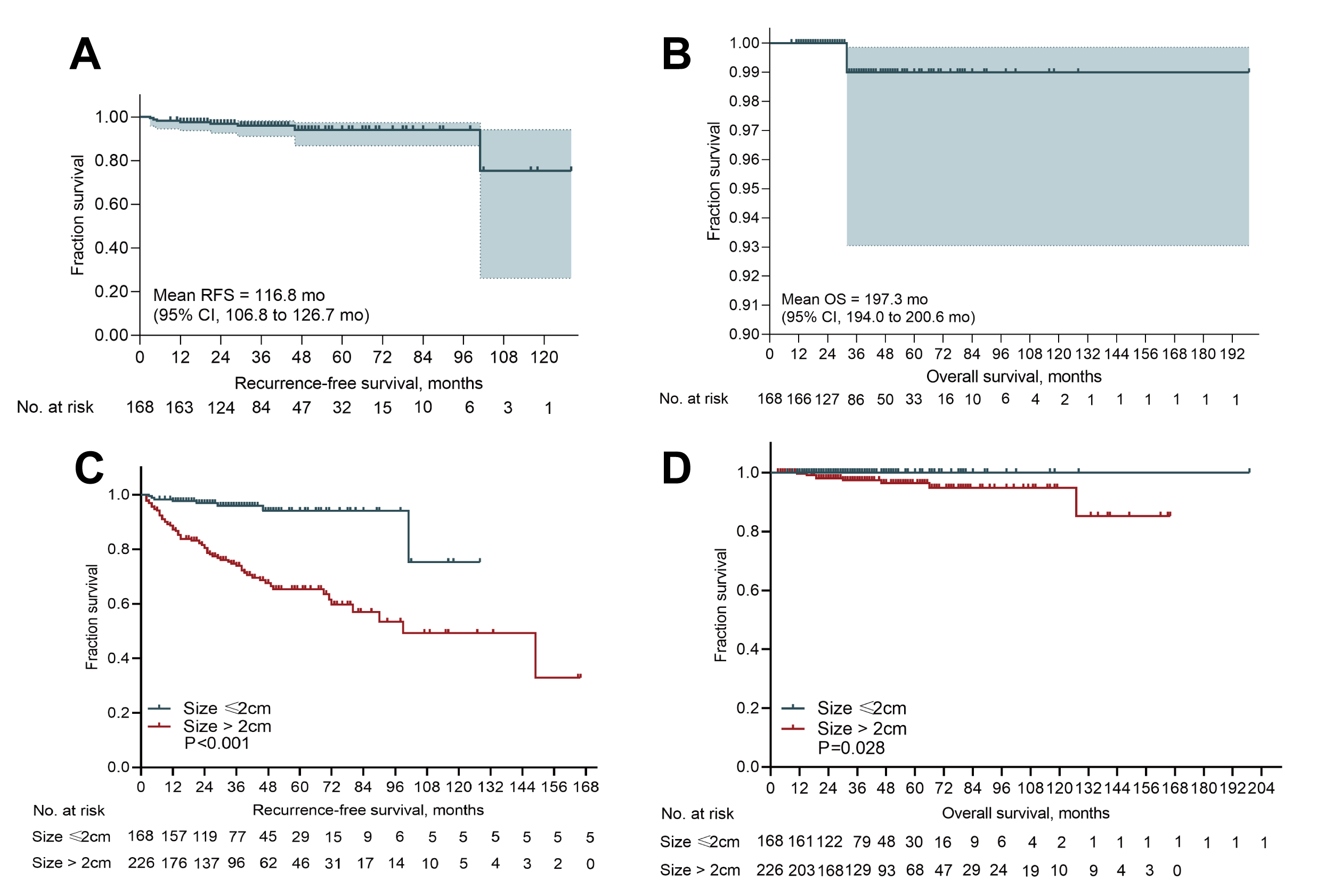

Supplement: Supplementary file 1 [file cancers-15-01955-s001.zip › Figure S1.tif]
